# An Investigation of Limbs Exercise as a Treatment in Improving the Psychomotor Speed in Older Adults with Mild Cognitive Impairment

**DOI:** 10.3390/brainsci9100277

**Published:** 2019-10-16

**Authors:** Hao Jiang, Shihui Chen, Lina Wang, Xiaolei Liu

**Affiliations:** 1 School of Humanities and Social Science, Chinese University of HK (Shenzhen), Shenzhen 518172, China; jianghao@cuhk.edu.cn; 2 Department of Kinesiology, Texas A & M University, Texarkana, TX 75503, USA; 3 Nursing Department of Medical College, Huzhou University, Huzhou 313000, China; 02474@zjhu.edu.cn; 4 Department of Traditional Sports, Beijing Sport University, Beijing 100084, China; liuxiaolei99@hotmail.com

**Keywords:** dementia and Alzheimer’s disease, mild cognitive impairment, psychomotor speed, limbs exercise

## Abstract

Objectives: This study investigated the effects of therapeutic structured limb exercises intended to improve psychomotor speed in older adults with mild cognitive impairment (MCI). Methods: Forty-four patients with mild cognitive impairment who met the inclusion criteria were selected and assigned randomly to either an experimental group (22 patients) or a control group (22 patients). The numbers of participants were selected based on the calculated sample effect size (*N* = 38). The study involved a 10-week intervention, in which participants completed structured limb exercises during 60-min training sessions delivered three times per week. Forty-one subjects completed the experimental programme. Scores in the Finger Tapping Test (FTT), Purdue Pegboard Test (PPT) and Montreal Cognitive Assessment (MoCA), along with electroencephalography (EEG) data, were collected before, during and after the intervention. The experimental and control groups were compared using repeated measures analysis of variance. Results: The patients with MCI in the experimental group achieved significantly improved scores in the FTT, the PPT and all dimensions of the MoCA. Moreover, these patients exhibited significant increases in the alpha and beta EEG wave power values in all brain areas of MCI patients, indicating that limb exercise training positively influenced their brain functions. Conclusions: The results conclude that a structured therapeutic limb exercise intervention can effectively improve psychomotor speed in patients with MCI and mitigate declines in cognitive function. This training intervention appears to be effective as a treatment for community-dwelling patients with MCI.

## 1. Introduction

Population ageing is a global phenomenon that has attracted international attention in the twenty-first century. Statistics indicate that older adults (aged >60 years) accounted for 11% of the global population in 2000, and estimates suggest that this population will double and thus account for 22% of the world population by 2050 [1]. Alzheimer’s disease (AD) is a chronic neurodegenerative disease that severely impairs a person’s cognitive function, thus hindering their self-care ability and inducing disordered psychological behaviour [2]. According to the WHO, approximately 35.6 million people were affected by dementia in 2012, representing an annual increase of 4.6 million cases. The number of AD patients is expected to double within decades if timely and effective preventive measures and interventions are not provided [3]. 

Mild cognitive impairment (MCI) is a transitional stage between normal brain ageing and early-stage dementia (particularly AD). Although people with MCI are affected by symptoms of memory impairment at an earlier than normal stage, their memory functions remain satisfactory and do not severely affect their daily lives or activities [4]. Older adults commonly experience decreases in cognitive function with ageing, and this trend is particularly evident with respect to memory function [5]. In the initial stage, AD is frequently associated with MCI and the gradual development of cognitive impairment and functional disorders within a few years [6]. Therefore, MCI accelerates the risk of AD development, and in particular, memory impairment is a major symptom in patients with MCI. In the absence of timely treatment, 50% of patients with MCI will develop AD within 5 years [7]. Current studies of AD prevention have shifted from the clinical phase to the preclinical phase, given the particular importance of MCI prevention and reduction in older adults (aged ≥65 years). In other words, early MCI diagnosis and intervention are critical to preventing the development of AD.

Psychomotor speed (PS) is a sensitive index used to measure psychomotor function [8]. PS tests are used to measure central brain functions, such as the reaction speed and accuracy, during the performance of simple or complicated actions after receiving stimulation. Early studies of PS measurement included only the simple reaction time and choice reaction time [9,10,11]. (Pate and Margolin, 1994; Baddeley et al., 2001; Levinoff et al., 2005). However, these parameters comprise only a fraction of PS behaviours. According to Sobin and Sackeim [12], who conducted an observation of the psychomotor characteristics of patients with depression, four levels of PS can be observed and measured: gross motor activity, body movements, speech and motor response time. A slow PS manifests in multiple forms, and the external signs mainly involve slowness in basic movements (e.g., whole body activity or fine motor activities), slowness and incoherence in language, and reduced calculation abilities. People with a severely slowed PS may experience a decline in their performance of daily activities (e.g., bathing, getting out of bed, dressing and answering phone calls) and may be incapable of making prompt decisions in response to unexpected incidents [13]. (MBF, 2008). Patients with MCI exhibit evident deficiencies in their abilities to perform dual tasks [14]. Moreover, a slow PS may be particularly evident in patients with mental illnesses, such as depression [15,16] or schizophrenia [17]. Studies have indicated that depression is a risk factor for the development of dementia and MCI, and that the cognitive function symptoms exhibited by patients with depression and MCI are highly consistent with the symptoms associated with a slow PS [18,19]. Therefore, this study used an improvement in PS as the basis for establishing an intervention strategy intended to delay and prevent continuous impairments in cognitive function in patients with MCI.

Recent studies have explored the critical ability of physical exercise to improve cognitive abilities and brain function. Numerous studies have verified the protective effect of physical exercise on cognitive function in later life [20,21], as well as the different effects of variation in the form, intensity and frequency of exercise [22,23]. The effects of an exercise intervention on the cognitive functions of young and older adults have been investigated by several studies. Levin, Leon, Karssemeijer and their teams [24,25,26] utilizes systematic review and meta-analysis to analyse the combined cognitive and physical exercises on cognitive function in older adults with MCI or dementia. The results of their studies reported positive cognitive benefits for older people with MCI or dementia. 

Opportune exercise training has critical effects on the cognitive functions of older adults. In older adults, physical condition is related closely to the degeneration of hippocampal brain tissue. Exercise can induce fibroblast growth factor expression in the hippocampus and increase the hippocampal volume in older adults. Consequently, these effects alter the brain structure, enhance neuronal vitality and delay the degeneration of cognitive function in this population. Therefore, exercise is clearly a valuable component of MCI prevention [27]. 

Currently, no treatments prevent or reverse the course of AD, and only a few options can be used to ease and improve symptoms temporarily [28]. MCI intervention strategies comprise both drug and non-drug interventions, such as physical training, cognitive interventions and psychological interventions. Most studies have recommended that MCI treatments focus primarily on non-drug interventions to prevent and reduce the side effects of drugs [29,30]. There are several studies that used psychomotor speed, processing speed and reaction time to measure the effects of physical training for the improvement of participants [31]. Pluncevic-Gligoroska [16] and Poon’ studies aimed to examine the possible relationship between psychomotor speed expressed by the Trial Making Test (TMT), the results suggest that regular physical activity have a positive impact on cognitive processes. Poon’s studies also conclude that the psychomotor speed of fit young and older adults is faster than the speeds of their less fit age-mates, and that physical training programs results in the improvement of participants’ response speeds. 

Although several studies have investigated the beneficial effects of different types of physical exercises (e.g., Tai Chi, bowling/croquet, cycling, resistance training, waling, psychomotor exercises and aerobic exercises) on psychomotor and cognitive functions in old age [24,25,32,33], previous studies have rarely reported using a structured therapeutic psychomotor limbs-exercise programme as a treatment on the effects of older adults with MCI who reside in urban communities and nursing homes. In addition, singular exercise training methods have been used in previous studies, and the conventional content designs of these methods have frequently neglected mind and body related exercises and PS, a sensitive indicator of cognitive function. This study had the following goals:

- To determine whether PS is correlated with cognition level in patients with MCI and whether it can be regarded as an indicator to measure the cognitive functions of patients with MCI;
- to assess a potential structured limbs exercise programme to improve the cognitive functions of MCI patients according to their PS and cognition level; and
- to evaluate a limb exercise project intended to improve the PS of patients with MCI to improve cognitive functions such as cognition level and electroencephalography (EEG) results.

## 2. Materials and Methods

### 2.1. Participants

Our screening of older adults residing in Communities A and B and Nursing Home C yielded 44 elderly patients with MCI. We divided the subjects randomly into a limb exercise training intervention group and a control group. The criteria for participant recruitment are shown in Table 1.

Sample Size Calculation: Prior to the data collection, power analysis was performed to determine the sample size. Based on our pilot study of the experimental design, we determined a large effect (Cohen’s *f* = 0.502) of the intervention on the cognition level (measured using the Montreal Cognitive Assessment (MoCA)). At an α value of 0.05 and β (power) value of 0.95, our power analysis indicated that 19 participants per group would be required to detect a large effect (*N* = 38). The decision was made to oversample (*N* = 44) to accommodate an expected attrition rate of 15%. The 44 subjects resided in 15 residential quarters of two urban communities. Equal numbers of patients were included in the control and experimental groups because classrooms were assigned randomly to the two groups (Table 2). A power analysis indicated that the sample size was adequate for the proposed study.

### 2.2. Instruments

We randomly selected 44 elderly participants aged 60–70 years. The Mini Mental State Examination (MMSE) and Activities of Daily Living (ADL) Scale were used to screen subjects for the study. The following test instruments were used to collect data at the baseline, interim and post-intervention time points and to verify the results of the intervention: for PS, the Finger Tapping Test (FTT) and Purdue Pegboard Test (PPT); and for cognitive level, MoCA and EEG (Emotive EPOC, Emotive Company, SF, USA.

### 2.3. Structured Limbs-Exercises

The limb exercise at this stage aims to develop the task execution capacity, attentiveness, fine motor skills, hand–eye coordination, reaction time, balance, and upper and lower body coordination of MCI patients (Table 3).

### 2.4. Experimental Design

This study adopted a quasi-experimental design that included both a randomised control trial and a questionnaire. Under this quasi-experimental design, the study applied grouped comparisons among the baseline, interim and post-test time points, and used a repeated measures analysis of variance (ANOVA) for the data analysis. Subjects were allocated randomly to the experimental and control groups. Details of the test arrangements are shown in Table 4.

### 2.5. Intervention and Procedures

Five patients with MCI who satisfied the recruitment criteria and passed a screening protocol based on the MMSE and ADL scales were included in a pilot study. This pilot study was conducted to improve the preliminary procedures at an early experimental stage, derive relevant assumptions and increase the rates of success of subsequent experiments in the official programme. The formal research intervention was commenced upon completing the pilot study.

During the formal experiment, the subjects in the control group participated in regular activities at a community senior activity centre (e.g., reading, chess and card games, singing and dancing). In addition to these regular daily activities, the subjects in the experimental group participated in a limb exercise intervention. They were divided into several subgroups (*n* = 7 or 8 per group). The duration of each limb exercise session was 60 min, and the sessions were held three times per week. During the first week, the researcher taught the limb exercise to the subjects. Beginning in the second week, subgroups of subjects were asked to participate in the limb exercise intervention. The subjects were also required to keep a record of their training conditions. The researcher and group heads monitored and instructed the subjects in each group. 

**Figure 1:**
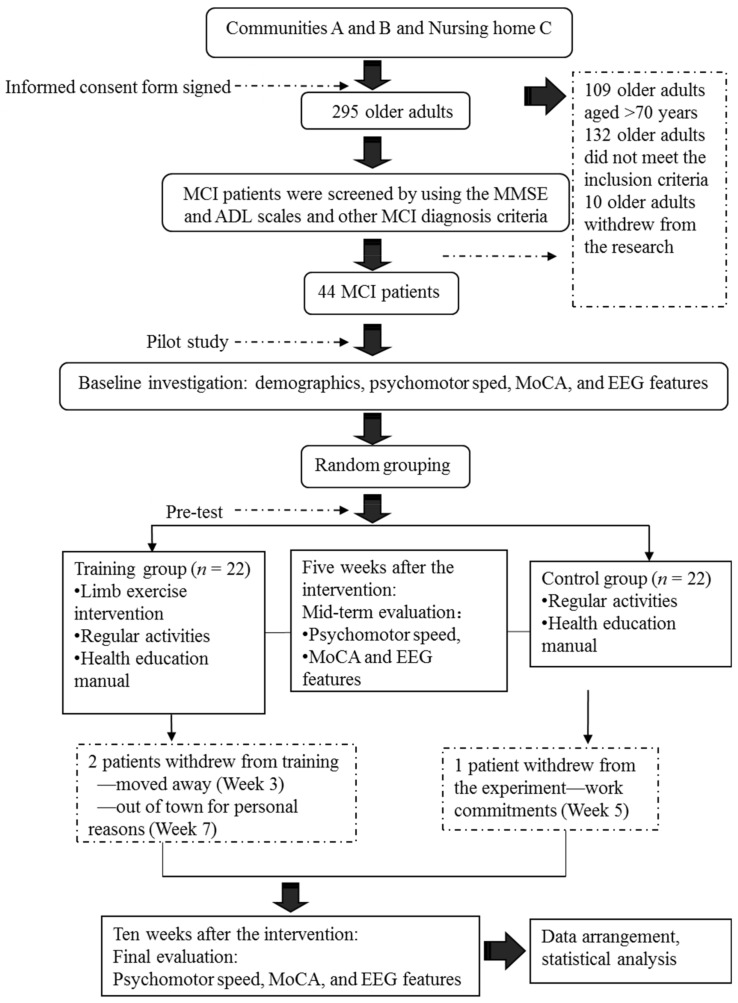
Flowchart of the research procedure.

The research intervention procedures are described in Figure 1.

### 2.6. Statistics and Data Analysis

SPSS Version 18 was used to organise and analyse the data. The χ^2^ test and an independent sample t-test were used to compare the baseline levels of all variables between the two groups. To evaluate the measurements and ranked data with non-normal distributions, the Mann–Whitney U rank-sum test was used to balance the distributions of the related variables between the groups before the intervention. Spearman’s rank correlation coefficient was used to investigate the relationship between the subjects’ PS and cognition levels.

Significant differences in the subjects’ PS, MoCA scores and EEG features were analysed to evaluate the influence of the limb exercise intervention on cognitive functions.

## 3. Results

### 3.1. Correlational Analysis of PS vs. Cognition Level

Spearman’s rank correlation coefficient was calculated for a correlational analysis of PS. As shown in Table 5, all of the FTT and PPT dimension scores were correlated significantly with each other and with the MoCA test scores (all *p* < 0.05). 

The PPT scores directly reflected the patients’ movement conditions and were correlated strongly with the ADLs and movement-related scores. The FTT was correlated strongly with some ADLs and was influenced mainly by the patient’s cognitive and psychomotor conditions. The results of this study confirm the correlation between the quantitative motor measure (QMM) and cognition level.

### 3.2. Typical EEG Characteristics Observed in MCI Patients

**Figure 2:**
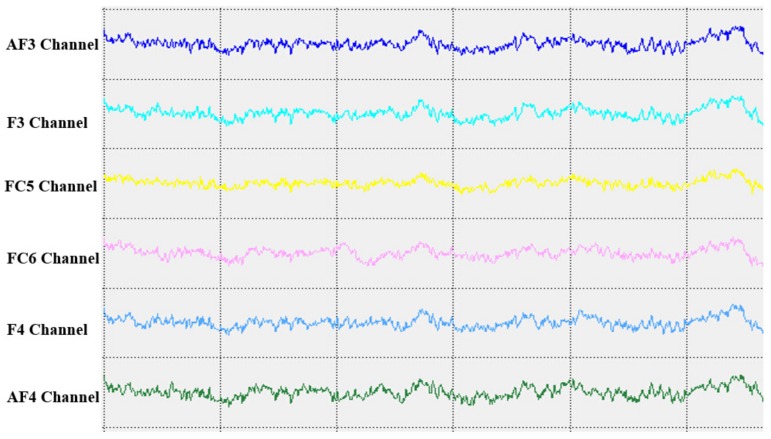
Six-channel electroencephalography (EEG) waveforms from a female subject.

The basic components of the EEG images obtained from 41 patients with MCI were examined and analysed. The measurements revealed a decline in alpha rhythm speed, alpha wave activity and alpha amplitude. Alpha wave activity slowed and even plateaued as the alpha index decreased. In comparison, beta activity decreased slightly, and theta activity increased moderately (Figure 2).

### 3.3. Verification of the Role of the Limb Exercise Intervention in Enhancing the Cognition Levels of Patients with MCI 

#### 3.3.1. Influence of the Limb Exercise Intervention on the PS of Patients with MCI 

Before the intervention, the researcher examined the PS of the research subjects via the FTT and PPT. On average, the subjects tapped their fingers 45.00 ± 5.43 times with the left hand and 48.39 ± 4.86 times with the right hand. No statistically significant differences were observed between the test results of the two groups (all *p* > 0.05). During the PPT, an average of 12.27 ± 1.80, 13.22 ± 1.93, and 10.75 ± 1.51 pegs were inserted correctly by the left hand, the right hand and both hands, respectively. No statistically significant differences were observed between the two groups (all *p* > 0.05). In other words, the subjects in the two test groups achieved balanced FTT and PPT results and were thus comparable (Table 6).

The PS of the research subjects in both groups was evaluated using the FTT and PPT at baseline and at Weeks 5 and 10. At the end of Week 10, the limb exercise group achieved higher scores in two dimensions of the FTT and three dimensions of the PPT relative to the baseline measurements. In comparison, the control group exhibited less substantial increases in the scores for the two dimensions of the FTT and three dimensions of the PPT. Table 7 and Table 8 present the FTT and PPT scores, respectively.

**Figure 3:**
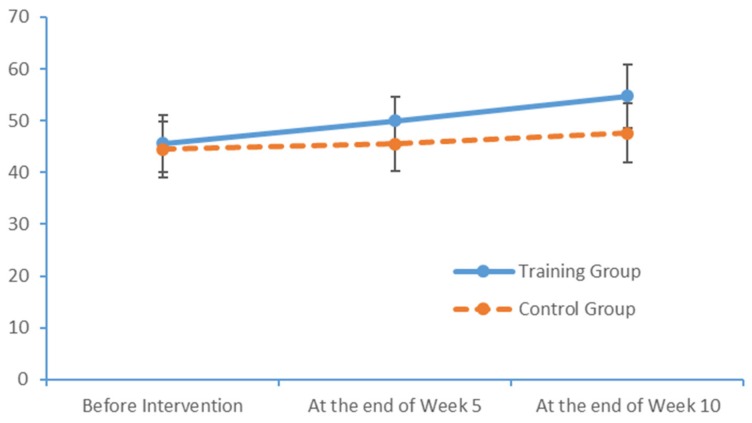
Changes in the left-hand FTT scores of the two groups at each time point.

**Figure 4:**
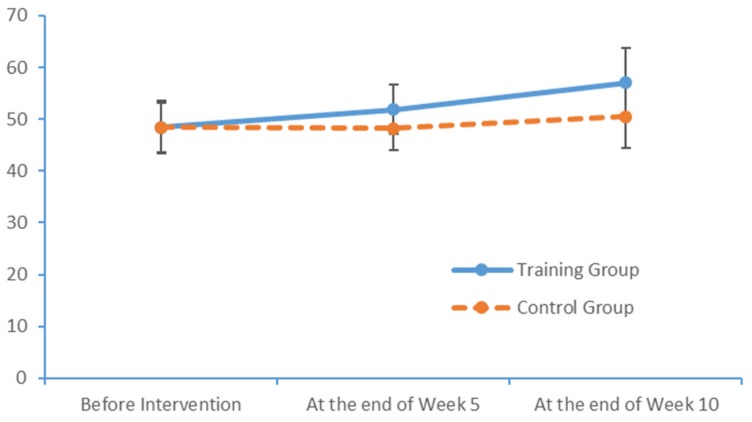
Changes in the right-hand FTT scores of the two groups at each time point.

Run charts were plotted to depict the indicators at the three measurement time points. Figure 3 and Figure 4 depict trends in the FTT scores of the two subject groups for the left and right hands, respectively. In particular, the training group exhibited more noticeable improvements over time.

**Figure 5:**
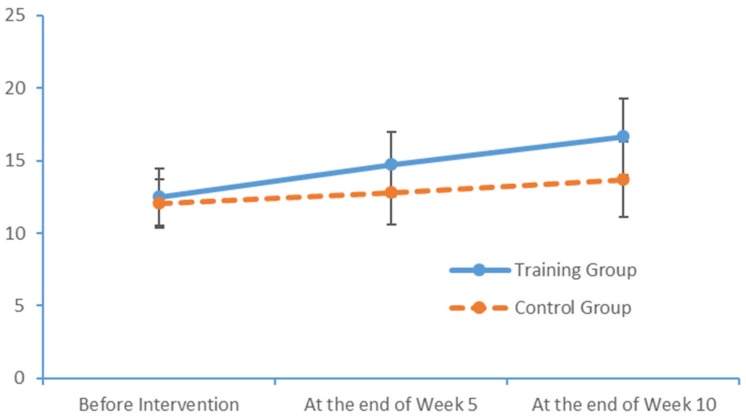
Changes in the left-hand PPT scores of the two groups at each time point.

**Figure 6:**
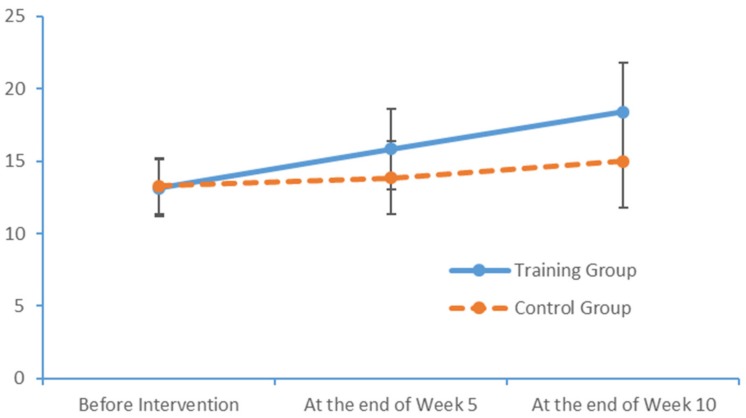
Changes in the right-hand PPT scores of the two groups at each time point.

**Figure 7:**
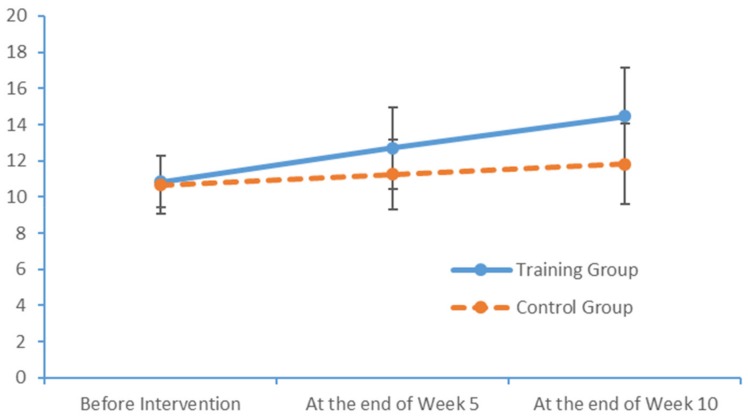
Changes in the left and right hands PPT scores of the two groups at each time point.

Run charts were also plotted to depict variations in the indicators measured at the three measurement times. Figure 5, Figure 6 and Figure 7 depict trends in the PPT scores of the two subject groups when the left hand, the right hand and both hands were tested. Again, the training group exhibited more noticeable improvements over time.

Influence of the limb exercise training intervention on the cognition levels of patients with MCI.

Before the intervention, the Beijing version of MoCA was employed to measure the subjects’ cognition level. The subjects achieved an average total score of 17.15 ± 4.53. As shown in Table 9, no significant differences in the visuospatial execution, naming, attention and calculation, language, abstraction, delayed recall and orientation scores were observed between the two groups (all *p* > 0.05).

The Beijing version of the MoCA was used to evaluate the cognition levels of both subject groups at baseline and Weeks 5 and 10. At Week 10, the subjects in the limb exercise training intervention group had higher MoCA scores than those measured at baseline. Although the control group also achieved increased MoCA scores, this increase was smaller than that observed in the experimental group (Table 10).

**Figure 8:**
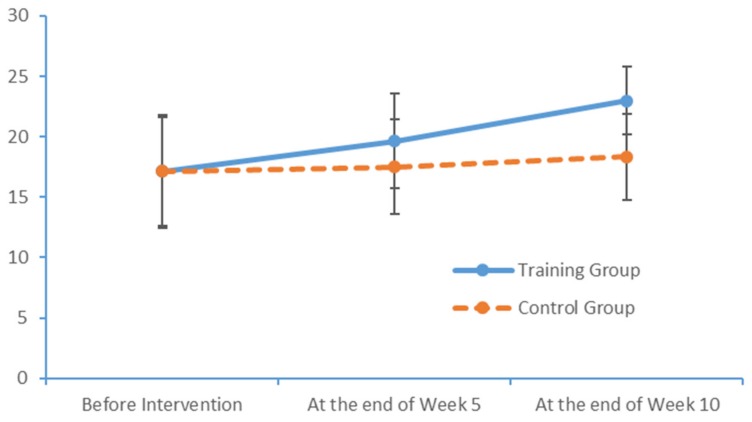
Changes in the MoCA scores of the two groups at each time point.

Run charts were then plotted for the indicators measured at the three time points. Figure 8 depicts the trends in the MoCA total scores of the two groups, revealing a more obvious improvement in the intervention group over time (Figure 8).

#### 3.3.2. Effect of Limb Exercise Training on the EEG Data Obtained from Patients with MCI

Before the intervention, the EEG data of the subjects in both groups were measured using an Emotiv EPOC neuroheadset. At baseline, no significant inter-group differences were observed in the alpha and beta wave power values in any brain areas (all *p* > 0.05), and both groups exhibited mostly consistent EEG power in all of the wavebands (Table 11). 

The alpha and beta EEG wavebands were again measured in the two subject groups at the ends of Weeks 5 and 10 of the intervention. The study further evaluated changes in the power values observed in all brain areas, as shown in Table 12.

**Figure 9:**
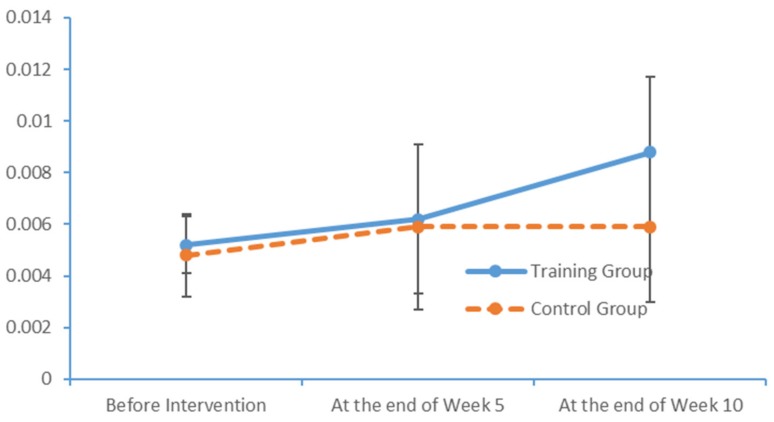
Changes in the power values of the alpha_hemi_left wavebands measured in the two subject groups at several time points.

**Figure 10:**
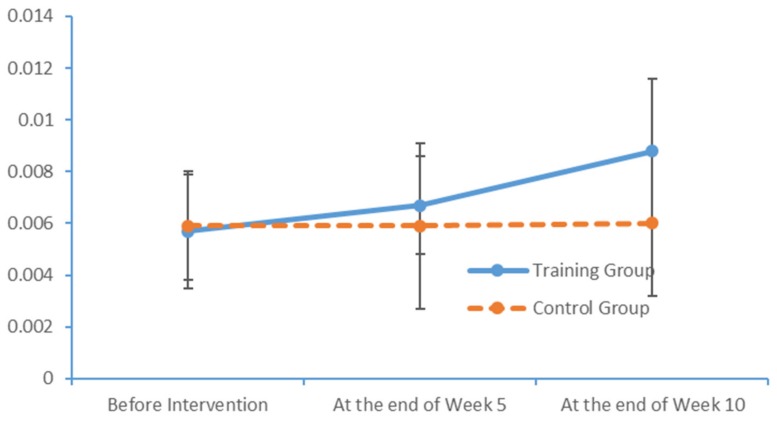
Changes in the power values of the alpha_hemi_right wavebands measured in the two subject groups at several time points.

**Figure 11:**
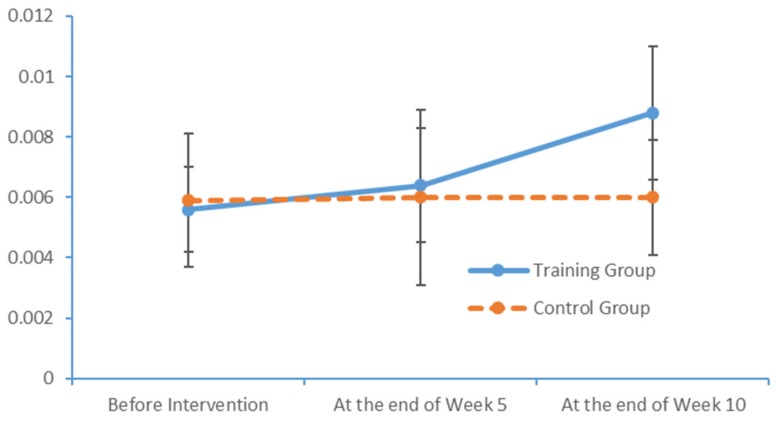
Changes in the power values of the alpha_region_front wavebands measured in the two subject groups at several time points.

**Figure 12:**
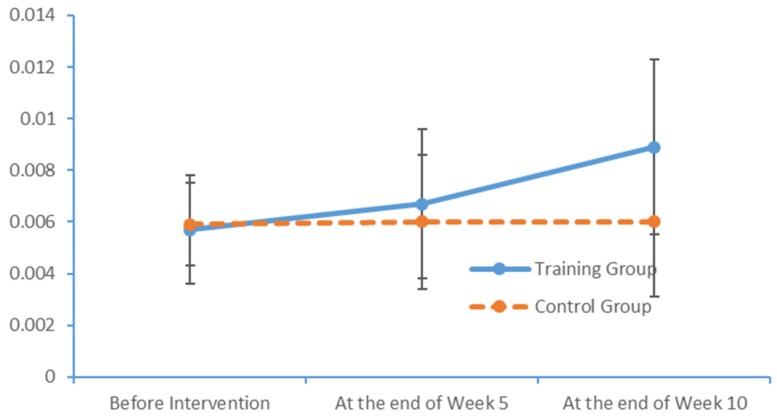
Changes in the power values of the alpha_region_central wavebands measured in the two subject groups at several time points.

Plot submenus were used to create run charts of the variations in the average indicator values, which were measured in triplicate. These run charts explicitly revealed changes in the alpha-wave power values in all brain regions over time. Figure 9, Figure 10, Figure 11 and Figure 12 demonstrate prominent variations in the data collected from the training group over time.

**Figure 13:**
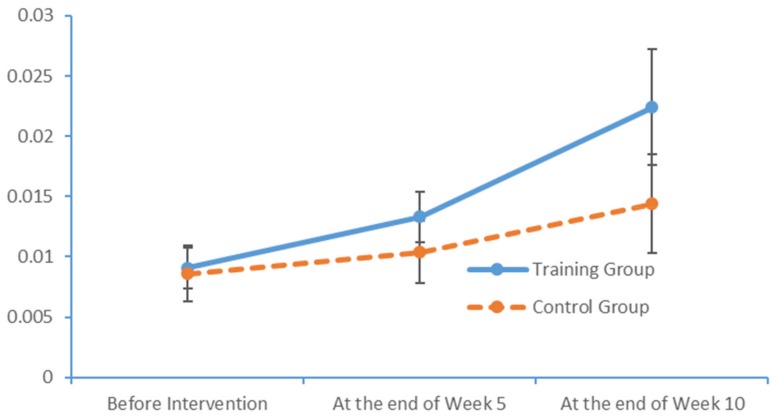
Changes in the power values of the beta_hemi_left wavebands measured in the two subject groups at several time points.

**Figure 14:**
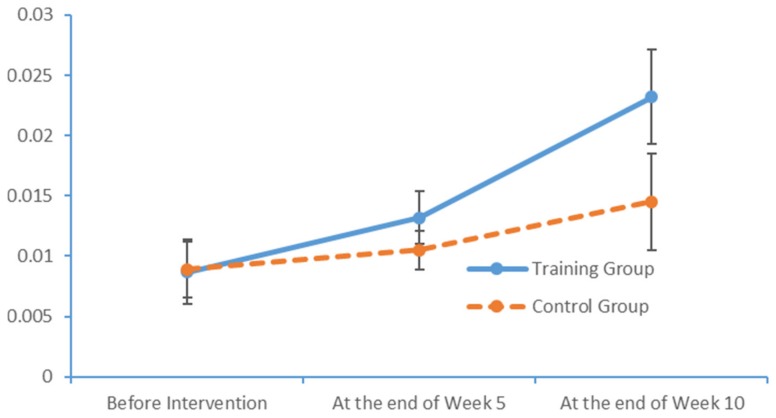
Changes in the power values of the beta_hemi_right wavebands measured in the two subject groups at several time points.

**Figure 15:**
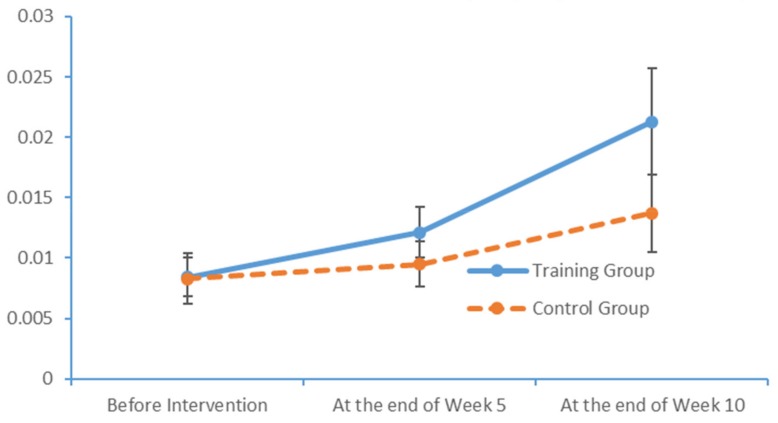
Changes in the power values of the beta_region_front wavebands measured in the two subject groups at several time points.

**Figure 16:**
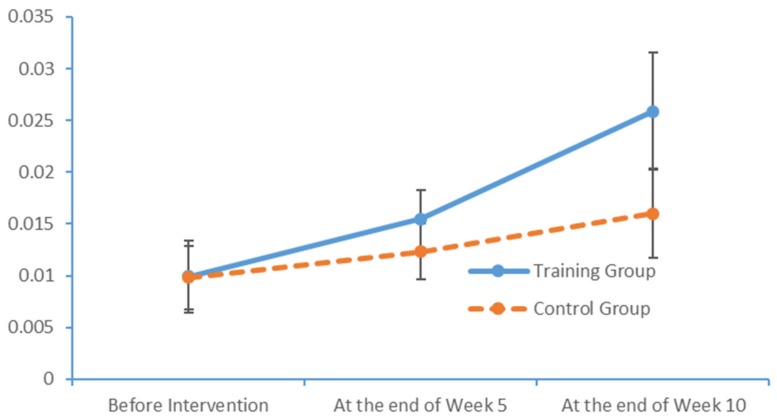
Changes in the power values of the beta_region_central wavebands measured in the two subject groups at several time points.

Plot submenus were then used to create run charts of variations in the average indicator values, which were also measured in triplicate. These run charts demonstrated changes in the beta-wave power values over time. Figure 13, Figure 14, Figure 15 and Figure 16 depict the substantial variations in data collected from the training group over time.

## 4. Discussion

In this study, we evaluated the forms, content and intensity of limb exercises designed to foster an increase in PS. The objectives of this study were to investigate the ability of these exercises to maintain the cognitive functions of patients with MCI, and to expose the effects of the exercises on cognitive functions in this patient population.

### 4.1. Correlation between the PSs and Cognitive Functions of Patients with MCI

In our study, the patients with MCI had mean finger tap frequencies of 45.00 for the left hand and 48.39 for the right hand, compared with an average of 37 per 10 seconds measured among healthy Chinese adults aged >45 years [31,32,33]. In other words, PS was far lower in the patients with MCI than in healthy adults. In the PPT, the subjects in our study achieved results similar to those of healthy older adults, as reported by Sun and Desrosiers [34,35]. That earlier study, however, did not test the cognitive functions of healthy elderly adults. Consequently, the PPT results of subjects in our study cannot yet be compared with those of healthy elderly adults with a normal level of cognitive function.

In our study, the MoCA scale scores revealed a relatively obvious decline in visuospatial and executive functions, consistent with the results of previously reported studies [36]. Most of the patients with MCI exhibited normal levels of ability with respect to place orientation but were unsure of the day of the week. This type of confusion might be due to the retired status of elderly adults and an associated lack of concern regarding the day of the week. In summary, our findings suggest that patients with MCI have accumulated damage in multiple cognitive domains, although the levels of damage vary between these domains. The most severe and prevalent damage was observed in the domains of delayed recall and visuospatial and executive function. An understanding of the characteristics of cognitive impairment in elderly adults with MCI could aid in the identification and diagnosis of community-dwelling patients, who could then be referred for an early-stage intervention. Moreover, our study results revealed significant correlations between the scores obtained in all dimensions of the FTT and PPT and all dimensions of the MoCA total score. Furthermore, the scores for all dimensions of the FTT were significantly correlated with those for all dimensions of the PPT. These results confirm that PS is a valid indicator of cognitive function and can be used for the preliminary screening of patients with MCI. 

The results may also support an analysis of the predictive value of the PS for the diagnosis of MCI. Therefore, an understanding of the characteristics of PS in elderly adults with MCI would assist in the early identification of affected patients, as well as early disease interventions.

### 4.2. Influences of Limb Exercise Training on the PS of Patients with MCI

During the limb exercise training project, the PPT and ball-holding exercises mainly targeted the fine motor control abilities of the hands, which indicate a person’s capacity to complete a specific task mainly via movements of the minor muscles in the hands and fingers in coordination with psychological activity (e.g., perception and attention). Previous studies [37] have investigated the influence of hand activities, such as clapping, forming and releasing a fist, and consecutive finger reversal with both hands, on cerebral cortex activation. Notably, clapping exerts the maximum effect on functions throughout the brain. Specifically, clapping induces maximum changes in the motion system and premotor areas, whereas consecutive finger reversal with both hands induces a maximum change in the prefrontal area. Activity in these brain areas is closely related to cognitive function. In this study, the fine motor abilities of patients with MCI were identified, and structured exercises were then designed to target fine motor training specifically. Statistical feedback from the FTT was examined and used to train the subjects with respect to the fine motor control abilities of the hands. This intervention profoundly prevented and mitigated the decline in cognitive function and fine motor ability in the hands observed in patients with MCI.

### 4.3. Influence of the Limb Exercise Training Intervention on the Cognition Levels of Patients with MCI 

After a 10-week limb exercise intervention, patients with MCI in our training intervention group exhibited an improvement of 5.85 points in the total MoCA score. Moreover, this improvement was statistically significant relative to the outcome of the control group. This study referenced the structured limb exercise interventions proposed by foreign scholars, Chinese cultural customs and the conditions of Chinese community- and nursing home-dwelling patients with MCI when designing the intervention. The results confirm that this limb exercise training intervention improved the cognitive functions of patients with MCI, consistent with the findings of several previous studies [38,39]. 

### 4.4. Effect of Limb Exercise Training on the EEG Activities of Patients with MCI

In the cerebral hemispheres, bioelectrical activities are triggered by cerebral cortical pyramidal cells and postsynaptic potentials and are regulated by nonspecific thalamic nuclei. The rhythms of EEG activity are affected by stimuli and feedback among the thalamus, brainstem reticular formation and cerebral cortex. Berger discovered that cerebral bioelectrical activities could be recorded via the scalp (www.epub.org). In a healthy adult, the EEG mainly comprises alpha and beta waves, of which the former comprises the majority. The two brainwaves are distributed in different areas of the brain. Alpha waves are distributed mainly in the occipital and parietal lobes, whereas beta waves are located mainly in the frontal and temporal lobes. The activities of alpha and beta waves are stable and change in response to pathological alterations in the brain. The run charts generated in this study explicitly revealed changes in the alpha wave power values in all brain regions over time, as well as prominent variations in the data collected from the training group over time. As patients with MCI face a high risk of AD, an understanding of the EEG characteristics in this population is highly valuable for MCI prevention.

### 4.5. Design of Limb Exercise Training Interventions Intended to Enhance the Health of Older Adults

Physical activity is critical to physical development in older adults. This type of activity can alter the social status and behaviour of this population and yield improvements in the quality of life. This study explored the physical and psychological development of older adults and used various theories and skills from physical training studies to provide education, consultation and training related to physical health knowledge and skills. Consequently, the intervention developed in this study helped the participants to cultivate regular exercise habits and improve their physical capacities and cognitive functions, while ensuring harmonious physical and psychological development.

For older adults, a combination of limb exercises and health-related activities enables the development of a comprehensive education system that further enhances conventional nursing skills and yields improvements in physical status, cognitive functioning and overall health quality. This type of intervention enables the development of training programmes from diverse perspectives (e.g., physical skills, cognition, behaviour and emotions) and the incorporation of numerous scientific subjects, including physical education, psychology, physiology and nutrition. 

Limb exercise training is a scientific approach in which nursing personnel are provided with pre-service training in the aspects of caring for older adults and integrating physical activities with health education. Nursing personnel who participate in this pre-service training must then apply the newly acquired training methods. Moreover, the inclusion of whole-body physical activities has increased the flexibility and creativity of these training methods. Although previous studies have not proposed alternative limb exercise training with the intent to improve the MCI conditions of older adults, our results suggest that nursing homes should continue to incorporate these limb exercises experimentally in combination with other physical activities intended to improve the PS of patients with MCI.

## 5. Conclusions

This study compared the outcomes of an intervention group of 20 patients with MCI who completed a 10-week limb exercise training programme and a control group of 21 patients who only received health education manuals. A baseline analysis of the 41 research subjects revealed a significant correlation between the PS, an effective indicator of cognitive impairment, and the level of cognition in the subject population. After a 10-week intervention, patients in the intervention group exhibited improvements in PS and cognition level relative to their conditions before the intervention. Compared with their counterparts in the control group, the patients in the intervention group exhibited more significant improvements in their FTT, PPT and MoCA total and dimension scores. Moreover, the patients in the intervention group exhibited significantly greater increases in the alpha and beta EEG wave power values in all brain areas, suggesting that limb exercise training had a positive effect on brain function. In summary, the limb exercise training intervention induced significant improvements in the PS of the patients with MCI while mitigating a decline in their cognitive function. Therefore, the limb exercise training intervention appears to be effective for community-dwelling patients with MCI.

## Figures and Tables

**Table 1:** Inclusion criteria and exclusion criteria.

Inclusion Criteria	Exclusion Criteria
The inclusion criteria were based broadly on the diagnosis criteria of mild cognitive impairment (MCI): Chief complaint of memory impairment by the patient, caretaker or informants. Aged 60–70 years. Educational attainment of elementary school or higher. Score 24–26 on the scale of Mini Mental State Examination (MMSE, Typically, people with an educational attainment of elementary school receive a score of >20 on the MMSE scale, and those with an educational attainment of junior high school or above receive a score of >24. Relatively normal level of self-care ability in performing activities of daily living (ADL), indicated by attaining a score of >60 on an ADL scale. No history of dementia according to the diagnosis of a psychiatrist Being informed of the research project and voluntarily consenting to participate.	The exclusion criteria were based broadly on the diagnosis criteria of MCI: Patients with vascular dementia, Parkinson disease, or any physical or mental disease-causing brain dysfunction. Patients who had undergone surgery within 1 year. Patients with impaired visual and hearing functions Patients with impaired heart function who have been restricted from exercising. Patients with a terminal disease and a life expectancy of less than 6 months.

**Table 2:** Number of subjects by community, gender and group.

Community			Group		Total
			Control	Experimental	
A	Gender	Female	4	6	10
		Male	7	5	12
	Total		11	11	22
B	Gender	Female	5	7	12
		Male	6	5	11
	Total		11	11	22
Total			22	22	44

**Table 3:** Structured Limbs Exercises.

**Upper Limb Exercises**	
Pegboard exercise	Subjects are required to insert small metal pegs into a prepared pegboard. Subsequently, they have to remove the pegs in a specific order. The task is repeated 3 times, with each time lasting for approximately 3 min (total about 10 min)
Sandbag throwing exercise	Subjects are asked to stand at a designated place and throw palm-sized sandbags at a 50 × 50 cm^2^ area approximately 1.5–2.0 m away from them. The subjects are required to retrieve the sandbags and throw them again for 10 times.
Ball-holding exercise	Basketball or ball with identical size is used for the seated exercise. First, the subjects are required to sit steadily on a chair and holding a ball in front of the chest with both hands. Subjects are asked to rotate to the left and right 10 times each (1 min). Raise the ball over their head and then return to the original pose. These exercises are repeated for 10 times (1 min). Finally, hold the ball with both hands in front of their chest and apply pressure to the ball by pressing their palms against it. The exercise is repeated for 10 times (1 min). The ball-holding exercise approximately lasted for 5 min.
**Lower Limb Exercises**	
Hopscotch exercise	Forty 30 × 30 cm squares were marked on the ground as 10 rows with each row comprising 4 squares. The squares in each row are randomly numbered from 1 to 4. The subjects are required to move in the order of the numbered squares (from 1 to 4) from Row 1 to 10. Completing this process is counted as one round. The task can repeat for several times as required. The total practice time is about 10 min.
Foot exercise	Subjects are asked to stand while shifting the body weight alternatively between their toes and heels. The subjects are required to practice the exercise several times within 5 min.

**Table 4:** Experimental design.

Group	Baseline	Mid-Point (Weeks 1–5)	Trial Completion (Weeks 6–10)
Experimental Group	Regular activities	Intervention *	Intervention *
Control Group	Regular activities	Regular activities Health education handbook Health education lecture	Regular activities Health education handbook Health education lecture

* **Intervention**: regular activities; health education handbook; health education lecture; 60-min of structured limbs exercise.

**Table 5:** Correlation analysis on the dimensional and total scores of Finger Tapping Test (FTT), Purdue Pegboard Test (PPT), and Montreal Cognitive Assessment (MoCA).

Item	FTT (Left)	FTT (Right)	Purdue Pegboard (Left)	Purdue Pegboard (Left)	Purdue Pegboard (Right)	Purdue Pegboard (Both)	Naming	Attention	Language	Abstraction	Delayed recall	Orientation	Total
FTT (left)	1.00												
FTT (right)	0.88 **	1.00											
Purdue pegboard (left)	0.96 **	0.83 **	1.00										
Purdue pegboard (right)	0.86 **	0.82 **	0.88 **	1.00									
Purdue pegboard (both)	0.73 **	0.60 *	0.78 **	0.81 **	1.00								
Visuospatial execution	0.83 **	0.78 **	0.83 **	0.91 **	0.75 **	1.00							
Naming	0.66 *	0.60 *	0.66 *	0.78 **	0.56 *	0.78 **	1.00 **						
Attention	0.74 **	0.77 **	0.80 **	0.84 **	0.64 *	0.81 **	0.74 **	1.00					
Language	0.53 *	0.49 *	0.57 *	0.66 *	0.50 *	0.57 *	0.65 *	0.52 *	1.00				
Abstraction	0.71 **	0.66 *	0.78 **	0.78 **	0.59 *	0.71 **	0.65 *	0.84 **	0.50 *	1.00			
Delayed recall	0.80 **	0.78 **	0.83 **	0.89 **	0.75 **	0.81 **	0.77 **	0.80 **	0.66 *	0.77 **	1.00		
Orientation	0.65 *	0.53 *	0.73 **	0.74 **	0.63 *	0.69 *	0.69 *	0.66 *	0.63 *	0.63 *	0.70 **	1.00	
Total	0.83 **	0.79 **	0.87 **	0.94 **	0.74 **	0.90 **	0.86 **	0.91 **	0.74 **	0.85 **	0.93 **	0.79 **	1.00

Note: * denotes *p* < 0.01, ** and *p* < 0.001.

**Table 6:** Psychomotor Speed (*N* = 41, and $\bar{X}$ ± SD).

Item	All Subjects	Training Group	Control Group	*t*-Value	*p*-Value
FTT					
FTT (left)	45.00 ± 5.43	45.60 ± 5.45	44.43 ± 5.48	0.686	0.497
FTT (right)	48.39 ± 4.86	48.40 ± 5.11	48.38 ± 4.72	0.012	0.990
PPT					
PPT (left)	12.27 ± 1.80	12.50 ± 1.96	12.05 ± 1.66	0.799	0.429
PPT (right)	13.22 ± 1.93	13.15 ± 1.98	13.29 ± 1.93	−0.222	0.825
PPT (both)	10.75 ±1.51	10.85 ± 1.42	10.67 ± 1.62	0.384	0.703

**Table 7:** FTT scores before and after intervention (*N* = 41, and $\bar{X}$ ± SD).

Item	Dimension	Baseline	Week 5	Week 10
Training group	FTT (left)	45.60 ± 5.45	49.95 ± 4.59	54.75 ± 6.19
	FTT (right)	48.40 ± 5.11	51.90 ± 4.75	57.05 ± 6.80
Control group	FTT (left)	44.43 ± 5.48	45.52 ± 5.15	47.62 ± 5.71
	FTT (right)	48.38 ± 4.72	48.19 ± 4.25	50.52 ± 6.02

**Table 8:** PPT scores before and after intervention (*N* = 41, and $\bar{X}$ ± SD).

Item	Dimension	Baseline	Week 5	Week 10
Training group	PPT (left)	12.50 ± 1.96	14.75 ± 2.24	16.65 ± 2.66
	PPT (right)	13.15 ± 1.98	15.85 ± 2.78	18.40 ± 3.41
	PPT (both)	10.85 ± 1.42	12.70 ± 2.25	14.45 ± 2.72
Control group	PPT (left)	12.05 ± 1.66	12.80 ± 2.18	13.71 ± 2.61
	PPT (right)	13.29 ± 1.93	13.86 ± 2.49	15.00 ± 3.21
	PPT (both)	10.67 ± 1.62	11.24 ± 1.92	11.81 ± 2.23

**Table 9:** Baseline cognition level (*N* = 41 and $\bar{X}$ ± SD).

Item	Research Subject	Training Group	Control Group	*t*-Value	*p*-Value
Visuospatial execution	2.66 ± 1.09	2.65 ± 1.14	2.67 ± 1.06	−0.048	0.962
Naming	2.37 ± 0.58	2.40 ± 0.50	2.33 ± 0.66	0.363	0.718
Attention and calculation	2.80 ± 0.78	2.80 ± 0.83	2.57 ± 0.67	0.967	0.967
Language	2.31 ± 0.65	2.25 ± 0.64	2.38 ± 0.67	−0.640	0.526
Abstraction	1.44 ± 0.63	1.45 ± 0.69	1.43 ± 0.59	0.107	0.916
Delayed recall	2.41 ± 0.92	2.35 ± 0.93	2.47 ± 0.92	−0.434	0.667
Orientation	3.07 ± 0.61	3.15 ± 0.67	3.00 ± 0.55	0.786	0.437
Total	17.15 ± 4.53	17.15 ± 4.67	17.14 ± 4.49	0.005	0.996

**Table 10:** MoCA scores at the different measurement times (*N* = 41, and $\bar{X}$ ± SD).

Item	Training Group			Control Group		
	Baseline	Week 5	Week 10	Baseline	Week 5	Week 10
visuospatial execution	2.65 ± 1.14	3.40 ± 1.05	3.85 ± 1.09	2.67 ± 1.06	2.86 ± 1.06	2.95 ± 1.12
Naming	2.40 ± 0.50	2.70 ± 0.66	3.55 ± 1.09	2.33 ± 0.66	2.52 ± 0.75	2.67 ± 0.86
Attention and calculation	2.80 ± 0.83	3.10 ± 1.07	3.60 ± 0.99	2.57 ± 0.67	2.67 ± 0.79	2.81 ± 0.81
Language	2.25 ± 0.64	2.65 ± 0.81	3.30 ± 0.73	2.38 ± 0.67	2.57 ± 0.59	2.76 ± 0.62
Abstraction	1.45 ± 0.69	1.70 ± 0.57	1.80 ± 0.62	1.43 ± 0.59	1.29 ± 0.56	1.29 ± 0.64
Delayed recall	2.35 ± 0.93	2.70 ± 1.03	3.25 ± 0.91	2.47 ± 0.92	2.57 ± 0.93	2.67 ± 0.79
Orientation	3.15 ± 0.67	3.35 ± 0.67	3.65 ± 0.59	3.00 ± 0.55	3.05 ± 0.67	3.19 ± 0.60
Total	17.15 ± 4.67	19.65 ± 3.92	23.00 ± 2.81	17.14 ± 4.49	17.52 ± 3.89	18.33 ± 3.59

**Table 11:** Comparison of the alpha- and beta-wave power in all brain areas (*N* = 41; $\bar{X}$ ± SD).

Brainwave Band	Research Subjects	Training Group	Control Group	*t*-Value	*p*-Value
alpha_hemi_left	0.0050 ± 0.0014	0.0052 ± 0.0011	0.0048 ± 0.0016	0.888	0.380
alpha_hemi_right	0.0058 ± 0.0022	0.0057 ± 0.0022	0.0059 ± 0.0021	−0.167	0.868
alpha_region_front	0.0058 ± 0.0019	0.0056 ± 0.0014	0.0059 ± 0.0022	−0.504	0.617
alpha_region_central	0.0058 ± 0.0019	0.0057 ± 0.0021	0.0059 ± 0.0016	−0.279	0.781
beta_hemi_left	0.0088 ± 0.0088	0.0091 ± 0.0017	0.0086 ± 0.0023	0.712	0.480
beta_hemi_right	0.0088 ± 0.0088	0.0087 ± 0.0027	0.0089 ± 0.0023	−0.276	0.784
beta_region_front	0.0083 ± 0.0083	0.0084 ± 0.0016	0.0083 ± 0.0021	0.169	0.867
beta_region_central	0.0098 ± 0.0099	0.0099 ± 0.0035	0.0098 ± 0.0031	0.133	0.895

**Table 12:** Pre- and post-intervention changes in the alpha- and beta-wave power of all brain areas of the subjects (*N* = 41; $\bar{X}$ ± SD).

Item	Training Group			Control Group		
	Baseline	Week 5	Week 10	Baseline	Week 5	Week 10
alpha_hemi_left	0.0052 ± 0.0011	0.0062 ± 0.0029	0.0088 ± 0.0029	0.0048 ± 0.0016	0.0059 ± 0.0032	0.0059 ± 0.0029
alpha_hemi_right	0.0057 ± 0.0022	0.0067 ± 0.0019	0.0088 ± 0.0028	0.0059 ± 0.0021	0.0059 ± 0.0032	0.0060 ± 0.0028
alpha_region_front	0.0056 ± 0.0014	0.0064 ± 0.0019	0.0088 ± 0.0022	0.0059 ± 0.0022	0.0060 ± 0.0029	0.0060 ± 0.0019
alpha_region_central	0.0057 ± 0.0021	0.0067 ± 0.0029	0.0089 ± 0.0034	0.0059 ± 0.0016	0.0060 ± 0.0026	0.0060 ± 0.0029
beta_hemi_left	0.0091 ± 0.0017	0.0133 ± 0.0021	0.0224 ± 0.0048	0.0086 ± 0.0023	0.0104 ± 0.0026	0.0144 ± 0.0041
beta_hemi_right	0.0087 ± 0.0027	0.0132 ± 0.0022	0.0232 ± 0.0039	0.0089 ± 0.0023	0.0105 ± 0.0016	0.0145 ± 0.0040
beta_region_front	0.0084 ± 0.0016	0.0121 ± 0.0021	0.0213 ± 0.0044	0.0083 ± 0.0021	0.0095 ± 0.0019	0.0137 ± 0.0032
beta_region_central	0.0099 ± 0.0035	0.0155 ± 0.0028	0.0259 ± 0.0057	0.0098 ± 0.0031	0.0123 ± 0.0027	0.0160 ± 0.0043
